# Evaluating iron deposition in gray matter nuclei of patients with acute ischemic stroke using quantitative susceptibility mapping

**DOI:** 10.3389/fneur.2024.1518911

**Published:** 2024-12-16

**Authors:** Li Zhou, Jie Yang, Wei Zhang, Limei Han, Shenghai Zhou, Chunyan Zheng, Hao Feng, Jianquan Zhong

**Affiliations:** Department of Radiology, Zigong First People's Hospital, Zigong, Sichuan, China

**Keywords:** acute ischemic stroke, brain iron, quantitative susceptibility mapping, gray matter nuclei, caudate nucleus, putamen

## Abstract

**Objectives:**

Understanding the microscopic pathophysiological mechanisms underlying acute ischemic stroke (AIS) is vital for facilitating early clinical diagnosis and intervention. In this study, we aimed to quantitatively assess brain iron changes in gray matter (GM) nuclei in patients with AIS via quantitative susceptibility mapping (QSM).

**Methods:**

Thirty-four patients with AIS and thirty age-and sex-matched healthy controls (HCs) were included. QSM and conventional magnetic resonance imaging were performed. Intergroup differences in regional susceptibility values were calculated for the bilateral caudate nucleus (CN), globus pallidus (GP), putamen (PUT), red nucleus (RN), substantia nigra (SN), thalamus (THA), and dentate nucleus (DN). A receiver operating characteristic curve was plotted to evaluate the classification and diagnostic performance of susceptibility values in distinguishing patients with AIS from HCs. Multiple linear regression analysis was used to investigate the impact of clinical variables on susceptibility values. Correlation analysis was used to assess the correlation between regional iron variations and clinical scores. A paired t test was used to calculate the differences in susceptibility values between the bilateral hemispheres in the participants.

**Results:**

Compared with the HCs, the patients with AIS had significantly increased susceptibility values in the bilateral CN and PUT (*p* < 0.05, FDR correction). The highest diagnostic performance was observed in the combination of susceptibility values with differences between groups (AUC = 0.722). Multiple linear regression analysis revealed that increased susceptibility in the right CN was significantly associated with smoking (*p* < 0.05). The susceptibility values were not significantly correlated with the clinical scores (*p* > 0.05), but age was positively correlated with the modified Rankin Scale scores at admission (*p* < 0.05). The susceptibility values of the SN exhibited lateral asymmetry in patients with AIS.

**Conclusion:**

This study revealed increased iron concentrations in the GM nuclei of patients with AIS. Iron deposition in GM nuclei may be a potential biomarker for further understanding the pathophysiological mechanism underlying AIS.

## 1 Introduction

Stroke is one of the leading causes of death and disability worldwide, imposing significant social and financial burdens (1). The stroke encompasses both ischemic and hemorrhagic stroke, with ischemic stroke accounting for approximately 85% of all cases (2). In the United States alone, nearly 800,000 people suffer a stroke annually, out of which around 700,000 are acute ischemic stroke (AIS) (3). AIS is characterized by high incidence rate, mortality rate, disability rate, and recurrence rate (5), necessitating advanced non-invasive magnetic resonance imaging (MRI) for further comprehension of its pathophysiological characteristics. When the ischemic stroke progresses to subacute stage, most ischemic lesions tend to evolve into a complete infarction and the fate of the ischemic lesion is basically determined (4).

The gray matter (GM) nuclei play an important role in motor control and cognitive function, and the normal metabolism of iron in the GM nuclei is essential for maintaining these functions. And then, the GM nuclei produce high levels of neurotransmitters involved in the information processing and present a progressive iron accumulation with age, so high levels of iron are exhibited in the GM nuclei (5, 6). Brain iron is a double-edged sword: on the one hand, it is crucial for various neurophysiological functions, such as oxygen binding and transportation, synthesis of neurotransmitter and protein, myelin production, and ATP production; on the other hand, it can also contribute to the development of diseases by causing reactive oxygen species production and oxidative stress (5, 7, 8). Quantitative susceptibility mapping (QSM) is one of the sophisticated processing methods for gradient-echo MRI, extensively employed for the quantification of the spatial distribution of magnetic susceptibility in biological tissues (9–11). Magnetic susceptibility refers to the response of magnetic materials in human tissue to an applied external magnetic field and different tissues show different susceptibilities (12, 13). It is currently believed that the susceptibility of GM is dominated by tissue iron, which is mainly stored in ferritin macromolecules (9). Ferritin complex is a kind of paramagnetic substance, indicating that iron increases the overall magnetic susceptibility of the tissue. Tissue susceptibility has been shown to have a positive linear relationship with iron concentration in deep GM nuclei (9, 14). The main source of magnetic susceptibility in GM nuclei has been reported to be iron, and quantitative evaluation of iron in these regions using QSM has emerged as a reliable non-invasive method (6, 9). Studies on QSM have reported excessive iron deposition in GM nuclei in various neurodegenerative diseases, such as Alzheimer’s disease, schizophrenia, Parkinson’s disease, and multiple sclerosis (15–21). Visualization and quantitative evaluation of changes in brain iron concentration could contribute to understand the underlying pathophysiological mechanisms of these diseases.

Ischemic stroke leads to hypoxic–ischemic of brain tissue, disrupting iron homeostasis and resulting in a surge of iron deposition in local brain tissue (22, 23). Ferroptosis is an iron-dependent and reactive oxygen species reliant cell death, which caused by massive lipid peroxidation mediated membrane damage (24). A study examined the model of unilateral, transient middle cerebral artery occlusion in rats and revealed an elevation of iron level within the lesioned hemisphere (25). Xia et al. reported the susceptibility value of the asymmetrically prominent cortical veins in the stroke hemisphere was significantly higher compared to that in the contralateral hemisphere and HCs (26). Hypertension is a prominent risk factor among stroke survivors, and the susceptibility value of subcortical structures in patients with hypertension was found to be significantly increased compared to that of healthy controls (HCs) (27, 28). Studies have found that patients with cerebral artery stenosis/occlusion showed significantly elevated susceptibility values in GM nuclei, which were found to be associated with several risk factors of cerebrovascular disease (23, 29). Previous studies have demonstrated that cerebral ischemia induces alterations of iron deposition in GM nuclei, which holds significant implications for comprehending pathological mechanism of cerebral ischemia; however, there remains a dearth of research on iron deposition resulting from AIS.

The aim of this study was to utilize QSM technology to identify alterations of iron accumulation in the GM nuclei among patients with AIS, and subsequently investigate its association with clinical scales. The identification of iron level abnormalities associated with early stage of ischemic stroke holds promise for informing clinical interventions and development of therapeutic drugs to enhance the neurological prognosis of patients.

## 2 Materials and methods

### 2.1 Participants

A total of thirty-four patients diagnosed with AIS and thirty HCs were recruited from Zigong First People Hospital. The following demographic and clinical information were collected: name, gender, age, race, risk factors of cerebrovascular disease (hypertension, hyperlipidemia, and diabetes), and time of onset, etc. In addition, the extent of neural function deficiency and prognosis of patients with AIS was assessed upon admission and discharge using the National Institutes of Health Stroke Scale (NIHSS) and modified Rankin Scale (mRS). This study was approved by the Ethics Committee and obtained informed consent from all participants.

The inclusion criteria of patients with AIS were as follows: (1) a diagnosis of AIS was established by at least two clinician and two radiologists based on comprehensive physical examination, neurological assessment, and MRI image; (2) the clinical diagnosis of AIS was based on the “Chinese Guidelines for the Diagnosis and Treatment of Acute Ischemic Stroke 2018”; (3) there were complete clinical data and good MRI image quality.

The exclusion criteria of patients with AIS were as follows: (1) history of severe neurological disorders, such as intracranial hemorrhage, brain tumor and brain surgery; (2) the brain infarction lesions affected region of interest (ROI) (bilateral caudate nucleus (CN), globus pallidus (GP), putamen (PUT), red nucleus (RN), substantia nigra (SN), thalamus (THA) and dentate nucleus (DN)); (3) unsuccessful MRI scans, due to factors such as the metallic implants or claustrophobia; (4) history of organic lesions in important organs.

The inclusion criteria for HCs were as follows: (1) no history of neuropsychiatric disorders; (2) no contraindications for MRI; (3) no history of severe organic lesions, such as cerebrovascular disease, brain injury, neurological disorders; (4) no history of alcohol and drug abuse.

### 2.2 Image acquisition

The complete image data, including QSM, T1-weighted imaging (T1WI), T2-weighted imaging (T2WI), and T2 fluid-attenuated inversion recovery (T2-FLAIR), diffusion weighted imaging (DWI) was collected on a 3.0 T MRI scan (MAGNETOM Vida; Siemens Healthineers, Erlangen, Germany), and the 64-channel head coil was selected for scanning.

QSM was conducted using an eight echo gradient-echo (GRE) sequence: repetition time (TR) = 55 ms, echo time (TE) = 6.15 ms, flip angle = 15°, field of view (FOV) = 220 × 220 mm^2^, thickness = 2 mm, number of slices = 72, voxel size = 0.9 × 0.9 × 2 mm^3^, acceleration mode: GRAPPA with 2X acceleration factor, and total scan time was 8 min 7 s.

### 2.3 Data analysis

#### 2.3.1 Data analysis of QSM

The QSM data were analyzed using the MEDI toolbox (Morphology Enabled Dipole Inversion) based on the Matlab 2018a software platform (Mathworks, Natick, MA, USA), following a structured workflow to ensure accurate and reliable results: (1) The image was unwrapped with Laplacian algorithm. (2) The FSL BET algorithm in MEDI toolbox was used to extract the brain mask. (3) The projection onto dipole fields (PDF) algorithm was used to remove the background field. (4) The susceptibility map was generated by MEDI local field inversion algorithm.

#### 2.3.2 Extracting the ROI

The susceptibility value of each ROI was quantified using ITK-SNAP by two radiologists who were blinded to the neurologic and clinical diagnosis. The ROIs included the bilateral CN, GP, PUT, RN, SN, THA, and DN. To enhance accuracy, the susceptibility value of each ROI was evaluated in three consecutive slices, and the average susceptibility value and standard deviation (SD) for each region were obtained.

### 2.4 Statistical analysis

SPSS 27.0 was used for statistical analysis. Independent t-test were used to compare the ages between patients with AIS and HCs. The χ2 test was used to compare gender data between the two groups.

The interclass correlation coefficient (ICC) was utilized to evaluate the interrater reliability of manual segmentation and the ICC ≥ 0.75 was considered excellent. The differences in corresponding ROI susceptibilities between patients with AIS and HCs were compared using either independent t-test or Mann–Whitney U tests, depending on the normal distribution of the data. The receiver operating characteristic (ROC) curve was plotted to evaluate the classification and diagnostic performance of the susceptibility values with significant differences between groups. Additionally, multiple linear regression analysis was used to investigate the impact of independent variables (demographic and clinical data) on the susceptibility values in GM nuclei. The correlation between regional iron variations and clinical scores were explored using either Pearson correlation or Spearman correlation test, depending on the normal distribution of the data. The paired t test was employed to calculate the differences of susceptibility values between the bilateral hemispheres in both patients with AIS and HCs. A value of *p* < 0.05 was considered to indicate statistical significance.

## 3 Results

### 3.1 Study population

A total of thirty-four patients with AIS (mean age: 71.32 years) and thirty HCs (mean age: 66.90 years) were included in this study. The demographics and clinical characteristics of patients with AIS and HCs are shown in Table 1. There were significant differences in the smoking history and hypertension history between the two groups (*p* < 0.001). There was no significant difference in other demographics or clinical characteristics between the two groups (*p* > 0.05).

**Table 1:** The demographic and clinical data of participants.

	Patients with AIS	Healthy controls	*p* value
	*n* = 34	*n* = 30	
Age (years)	71.32 ± 10.10	66.90 ± 7.81	0.057
Gender (male/female)	19/15	11/19	0.124
Smoking history (yes/no)	18/16	3/27	<0.001*
Alcohol history (yes/no)	10/24	3/27	0.054
Hypertension (yes/no)	24/10	0/30	<0.001*
Hyperlipidemia (yes/no)	5/29	3/27	0.570
Diabetes (yes/no)	7/27	2/28	0.110
NIHSS score at admission	2.0 (4.25)	-	-
NIHSS score at discharge	1.0 (2.25)	-	-
mRS score at admission	2.0 (2.0)	-	-
mRS score at discharge	1.0 (1.25)	-	-

*p < 0.05; −, not applicable; values are given in means ± standard deviations or median (interquartile range). AIS, acute ischemic strokes; mRS, modified Rankin Scale; NIHSS, National Institutes of Health Stroke Scale.

### 3.2 Comparisons of susceptibility values between groups

**Figure 1:**
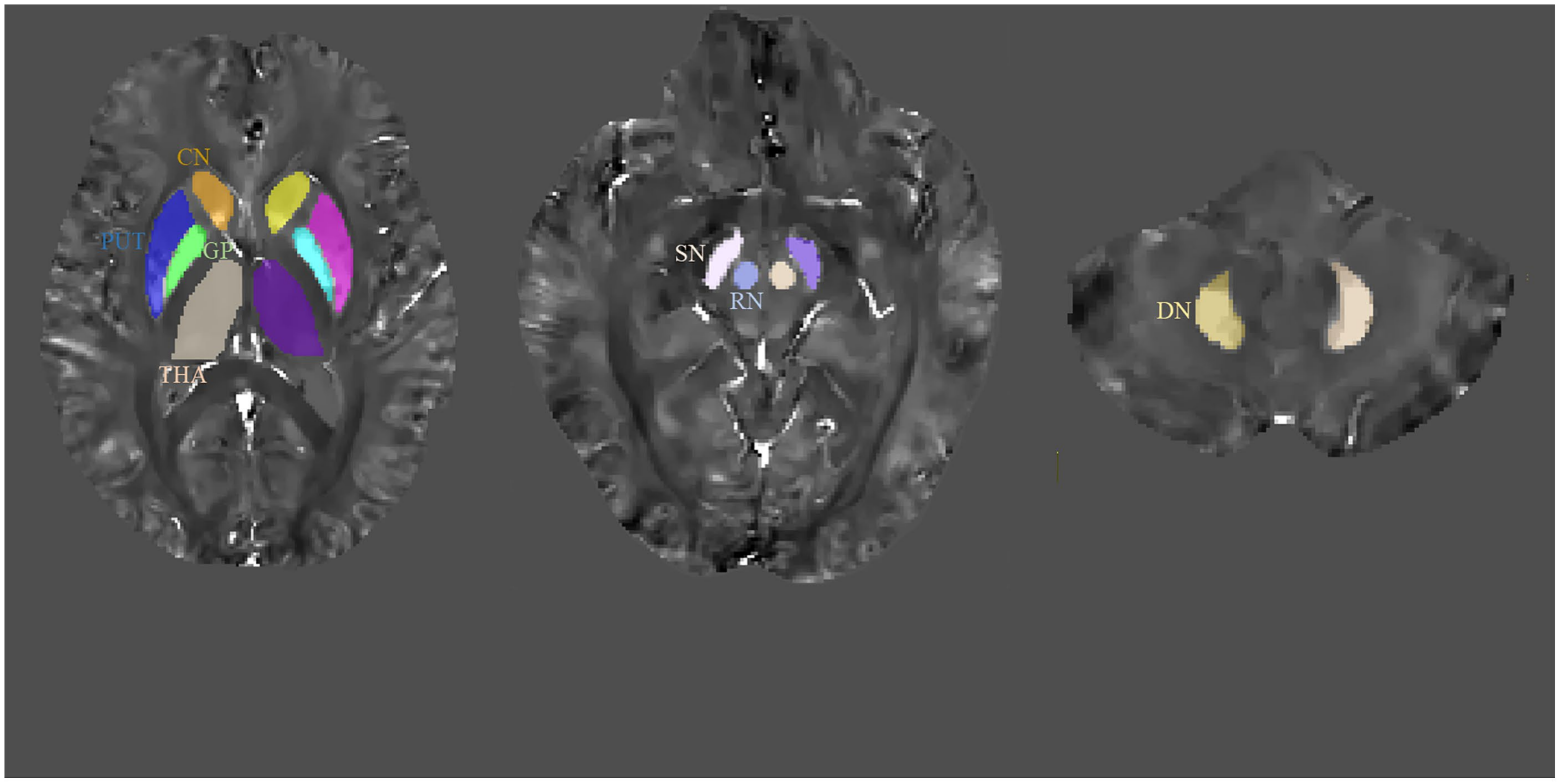
Regions-of-interest (ROIs) depicted on quantitative susceptibility mapping (QSM) images. CN, caudate nucleus; GP, globus pallidus; PUT, putamen; RN, red nucleus; SN, substantia nigra; THA, thalamus; DN, dentate nucleus.

The representative axial QSM sections are shown in Figure 1. The boundary of GM nuclei can be clearly seen on the QSM images, thereby demonstrating the feasibility of direct manual segmentation. The results of ICC analysis revealed excellent absolute agreement between raters in all segmented GM nuclei (0.823 ≤ ICCs ≤0.981, Table 2).

**Table 2:** Absolute ICCs for agreement between the raters for segmentation of the gray matter nuclei in patients with AIS and healthy controls.

		CN		GP		PUT		RN		SN		THA		DN	
		Left	Right	Left	Right	Left	Right	Left	Right	Left	Right	Left	Right	Left	Right
ICC	Patients with AIS	0.939	0.953	0.978	0.961	0.86	0.905	0.923	0.922	0.945	0.89	0.959	0.955	0.944	0.956
	Healthy controls	0.823	0.905	0.907	0.981	0.958	0.902	0.895	0.882	0.917	0.917	0.946	0.932	0.962	0.959

AIS, acute ischemic strokes; CN, caudate nucleus; DN, dentate nucleus; GP, globus pallidus; ICC, intraclass correlation coefficient; PUT, putamen; RN, red nucleus; ROI, region of interest; SN, substantia nigra; THA, thalamus.

**Figure 2:**
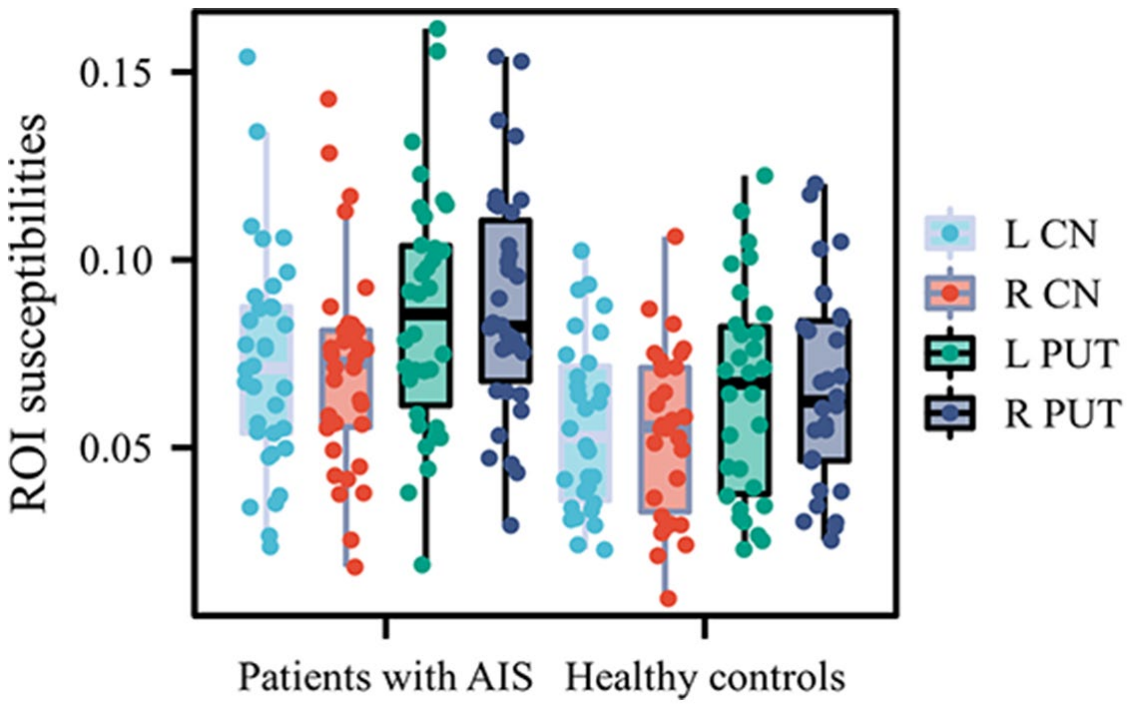
Quantitative comparison of susceptibility values in bilateral CN and PUT between patients with AIS with and healthy controls. CN, caudate nucleus; PUT, putamen; AIS, acute ischemic stroke.

The susceptibility values in bilateral CN and PUT were significantly increased in patients with AIS compared to HCs (susceptibility values in left CN: 0.07300 ± 0.02882 vs. 0.05570 ± 0.02277; susceptibility values in right CN: 0.07076 ± 0.02737 vs. 0.05405 ± 0.02256; susceptibility values in left PUT: 0.08580 ± 0.03239 vs. 0.06429 ± 0.02839; susceptibility values in right PUT: 0.08828 ± 0.03053 vs. 0.06531 ± 0.02670; *p* < 0.05, FDR corrected). The susceptibility values of other GM nuclei (bilateral GP, RN, SN, THA and DN) did not exhibit significantly different between the two groups (Table 3; Figure 2).

**Table 3:** ROI susceptibilities of the patients with AIS and healthy controls.

		Patients with AIS	Healthy controls	*P-FDR* value
CN	Left	0.07300 ± 0.02882	0.05570 ± 0.02277	0.042*
	Right	0.07076 ± 0.02737	0.05405 ± 0.02256	0.047*
GP	Left	0.17203 ± 0.05722	0.14285 (0.08172)	0.729
	Right	0.17960 ± 0.06094	0.15132 (0.06268)	0.564
PUT	Left	0.08580 ± 0.03239	0.06429 ± 0.02839	0.049*
	Right	0.08828 ± 0.03053	0.06531 ± 0.02670	0.028*
RN	Left	0.13421 ± 0.04802	0.12057 ± 0.03732	0.375
	Right	0.13429 ± 0.04533	0.12074 ± 0.03871	0.412
SN	Left	0.13194 (0.08458)	0.11392 (0.05587)	0.438
	Right	0.14977 ± 0.05966	0.13040 ± 0.04453	0.423
THA	Left	−0.00198 ± 0.01035	−0.00350 ± 0.00724	0.586
	Right	−0.00274 ± 0.00959	−0.00207 ± 0.00774	0.760
DN	Left	0.09596 (0.04371)	0.08232 (0.04186)	0.448
	Right	0.09071 (0.04433)	0.08779 ± 0.04074	0.467

*p < 0.05, FDR corrected; values are given in means ± standard deviations or median (interquartile range). AIS, acute ischemic strokes; CN, caudate nucleus; DN, dentate nucleus; GP, globus pallidus; PUT, putamen; RN, red nucleus; ROI, region of interest; SN, substantia nigra; THA, thalamus.

### 3.3 ROC curve analysis

**Figure 3:**
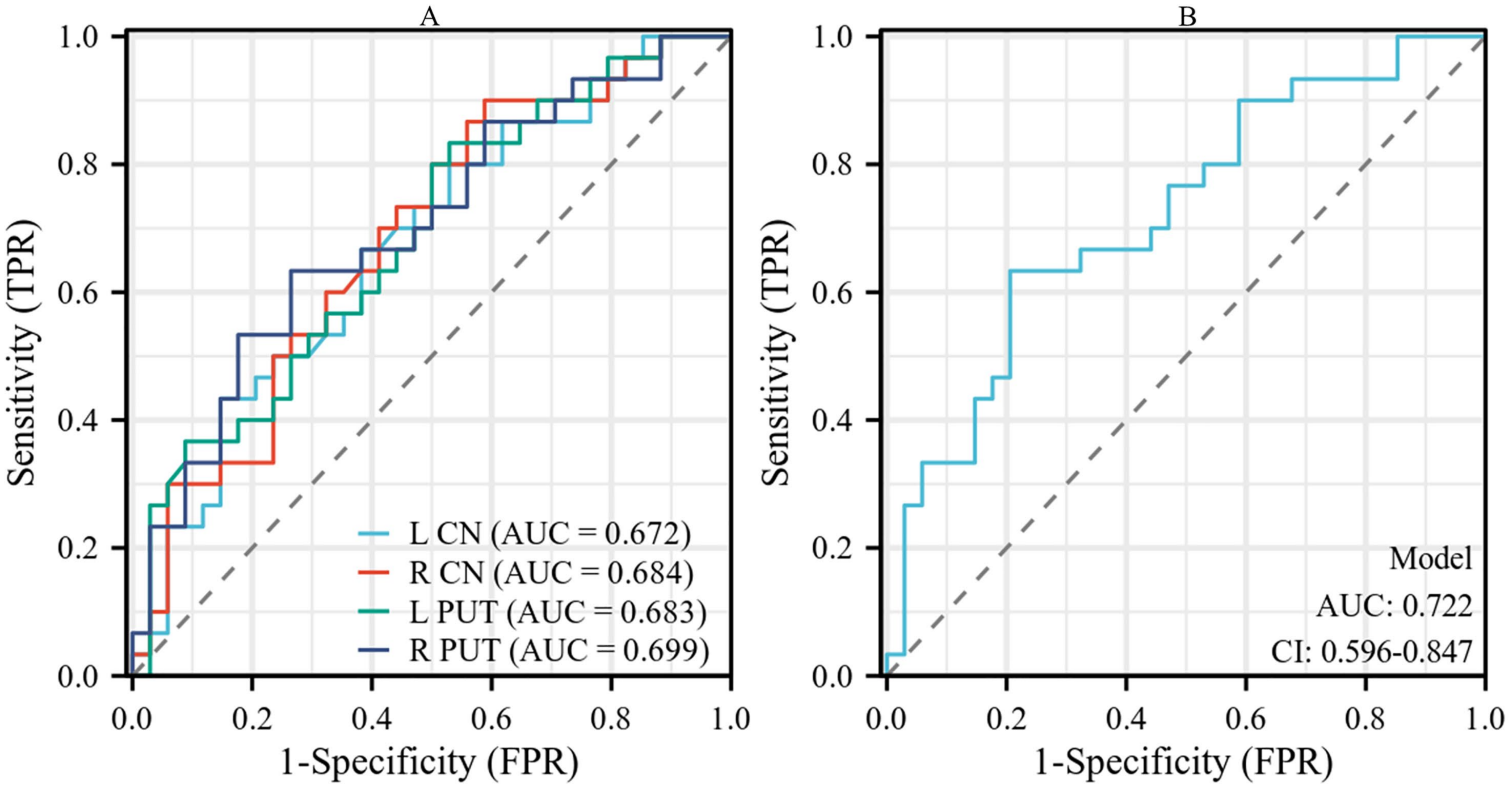
**(A)** Performance of susceptibility values of the bilateral CN and PUT in the diagnosis of patients with AIS. **(B)** The combined analysis of susceptibility values of bilateral CN and PUT showed a certain improvement in diagnostic performance. CN, caudate nucleus; PUT, putamen; AIS, acute ischemic stroke.

The results of the ROC curve analysis revealed that susceptibility values in the bilateral CN and PUT had some effect in distinguishing patients with AIS from HCs. The combination of susceptibility values in the bilateral CN and PUT showed better classification and diagnostic performance (AUC = 0.722) (Table 4; Figure 3).

**Table 4:** Diagnostic performance of ROI susceptibilities in patients with AIS and healthy controls.

Indicators		AUC	95%CI	Sensitivity	Specificity	Accuracy
CN	Left	0.672	0.538–0.805	0.433	0.853	0.656
	Right	0.684	0.552–0.815	0.900	0.412	0.641
PUT	Left	0.683	0.552–0.814	0.833	0.471	0.641
	Right	0.699	0.569–0.829	0.633	0.735	0.688
Combination of above indicators		0.722	0.569–0.847	0.633	0.794	0.719

AIS, acute ischemic strokes; AUC, area under the curve; CI, confidence interval; CN, caudate nucleus; PUT, putamen.

### 3.4 Multivariable linear regression analysis

Due to the relatively small sample size, variables with *p* values <0.1 in the univariate analysis were selected as independent variables for subsequent multiple linear regression analysis (Table 5). The results of multiple linear regression analysis showed that patients with smoking exhibited higher susceptibility values in the right CN (*p* = 0.012) (Table 6).

**Table 5:** Summary of univariate analysis linking demographic and clinical data with susceptibility values in the bilateral CN and PUT.

	L CN		R CN		L PUT		R PUT	
	*β*	*P*	*β*	*P*	*β*	*P*	*β*	*P*
Age	−0.043	0.809	−0.055	0.791	−0.029	0.869	−0.043	0.809
Smoking	−0.329	0.057*	−0.425	0.012*	−0.405	0.017*	−0.323	0.062*
Drinking	−0.131	0.460	−0.200	0.257	−0.402	0.018*	−0.272	0.120
Hypertension	−0.137	0.440	−0.196	0.267	−0.156	0.379	−0.138	0.437
Hyperlipidemia	−0.112	0.528	−0.146	0.409	−0.215	0.222	−0.136	0.441
Diabetes	−0.210	0.234	−0.221	0.210	−0.21	0.233	−0.278	0.111
NIHSS score at admission	0.194	0.314	0.165	0.394	0.364	0.052*	0.355	0.058*
NIHSS score at discharge	−0.090	0.662	−0.094	0.648	0.160	0.435	0.07	0.734
mRS score at admission	0.065	0.738	0.040	0.836	0.217	0.259	0.152	0.432
mRS score at discharge	−0.055	0.791	−0.094	0.646	0.074	0.719	0.095	0.645

*p < 0.1. CN, caudate nucleus; L, left; PUT, putamen; R, right.

**Table 6:** Summary of multivariable linear regression models linking demographic and clinical data with susceptibility values in the bilateral CN and PUT.

	Univariate analysis	*β*	*t*	*P*	Adjusted R^2^ of model	*F of model*	*P* of model
L CN	Smoking	−0.329	−1.973	0.057	0.081	3.895	0.057
R CN	Smoking	−0.425	−2.658	0.012	0.155	7.064	0.012*
L PUT	Smoking	−0.303	−1.44	0.162	0.168	2.887	0.056
	Drinking	−0.087	−0.377	0.709			
	NIHSS score at admission	0.299	1.557	0.132			
R PUT	Smoking	−0.106	−0.529	0.601	0.069	2.040	0.150
	NIHSS score at admission	0.312	1.558	0.131			

*p < 0.05. CN, caudate nucleus; L, left; PUT, putamen; R, right.

### 3.5 Correlation analysis

**Figure 4:**
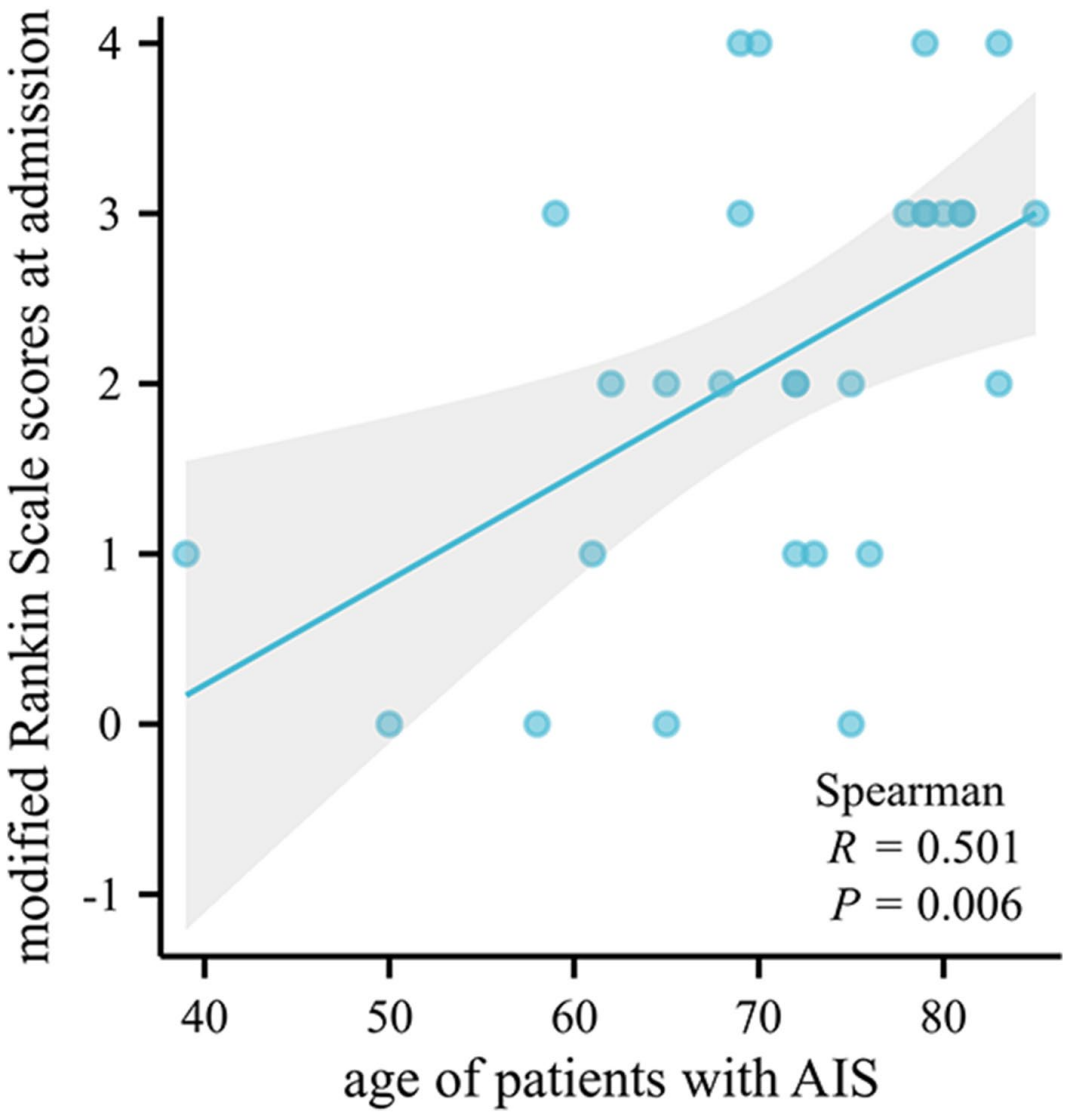
Bivariate scatter plots showing a positive correlation between the age of patients with AIS and the modified Rankin Scale scores at admission. AIS, acute ischemic stroke.

There was no statistically significant correlation between susceptibility values and clinical scores (*p* > 0.05), but the age of patients with AIS was positively correlated with the modified Rankin Scale scores at admission (*p* = 0.006, *r* = 0.501) (Figure 4).

### 3.6 The asymmetry of susceptibility values

**Figure 5:**
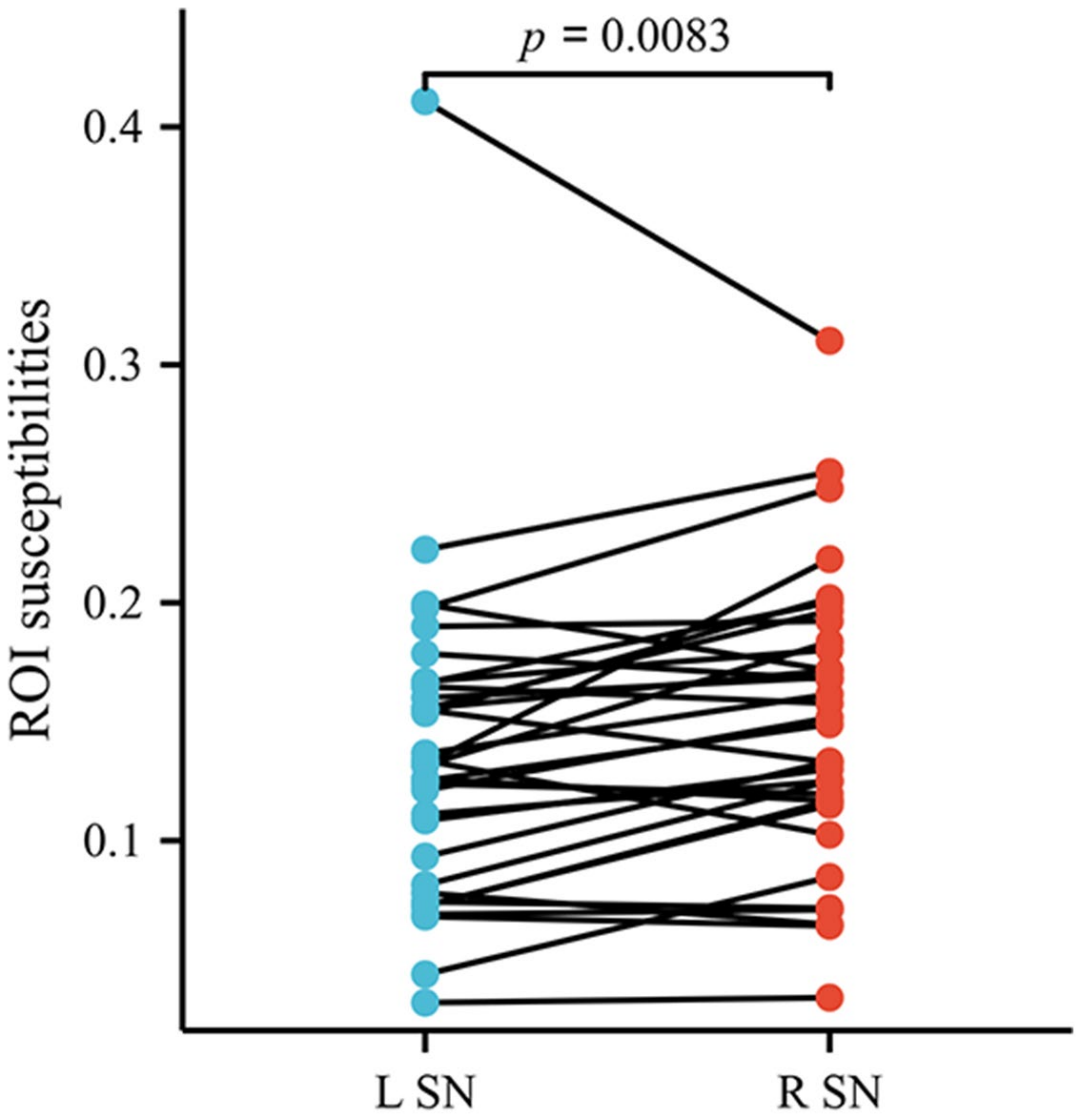
The susceptibility values of SN exhibited lateral asymmetry in patients with AIS. SN, substantia nigra; AIS, acute ischemic stroke.

The left–right asymmetry in the susceptibility values of SN was observed in patients with AIS by the paired t-test (the right side higher than left, susceptibility values in right SN = 0.149768 ± 0.059665, susceptibility values in left SN = 0.135634 ± 0.067580, *p* = 0.0083) (Figure 5). Comparable levels of susceptibility values were observed in other GM nuclei across bilateral hemispheres in patients with AIS. Susceptibility values of all ROIs in HCs did not exhibit left–right asymmetry.

## 4 Discussion

In the study, we utilized the QSM method to quantitatively compare iron concentrations in GM nuclei between patients with AIS and HCs. The results showed a significant increase in susceptibility values in bilateral CN and PUT in patients with AIS. Susceptibility values in bilateral CN and PUT had some effect in distinguishing patients with AIS from HCs. The result of multiple linear regression analysis indicate that smoking may have an impact on susceptibility values in CN in patients with AIS. The results of correlation analysis suggest that older patients with AIS may have worse neurological recovery outcomes. The susceptibility values of SN exhibited lateral asymmetry in patients with AIS, suggesting that alterations of iron level may not necessarily be paralleled between bilateral cerebral hemispheres.

The blood–brain barrier (BBB) plays a crucial role in regulating the physiological transport and metabolism of brain iron. The impairment of BBB endothelial cells can severely affect the normal iron absorption in hypoxic conditions that accompany AIS, leading to the release of excessive iron into brain tissue (22, 23). Iron overload can enhance Fenton reactions known as Haber-Weiss reaction (Fe^++^ + H_2_O_2_ → Fe^+++^ + OH^•^ + OH^−^), leading to an excessive production of hydroxyl (OH), and the highly reactive free radicals have the potential to cause damage to DNA, proteins, and lipids (30–32). Lipids play a crucial role in the cellular structure and function (such as cell membranes), and oxidative destruction of the lipids can ultimately lead to cytotoxic edema and subsequent cellular demise (29, 33). And then, aberrations in iron homeostasis *in vivo* are also associated with predisposing factors for ischemic stroke, such as cardiovascular disease, diabetes mellitus, hyperlipidemia, and hypertension (34–37). Disturbances of brain iron homeostasis may be involved in pathogenesis and progression of cerebral ischemia and stroke.

The findings of the study indicate that iron content in bilateral CN and PUT increases in patients with AIS, indicating alterations of microscopic and molecular properties in GM nuclei. Result of the ROC analysis showed that susceptibility values in bilateral CN and PUT could help us to distinguish patients with AIS from HCs. Similar to our finding, Mao et al. found that patients with middle cerebral artery stenosis/occlusion showed increased iron deposition in the PUT, GP, and SN, and the susceptibility values of GM nuclei exhibited correlations with risk factor variables for cerebrovascular disease (29). Du et al. revealed significant alterations in the susceptibility levels of GM nuclei in patients with middle cerebral artery occlusion, suggesting a potential involvement of iron metabolism disorder in the pathophysiological mechanisms underlying cerebrovascular disease (23). A study found that iron content in PUT and GP in patients with long-term cerebral ischemia significantly increased compared to HCs, which is consistent with our findings (38). The susceptibility values of infarct regions in patients with AIS were found to exhibit a significant increase compared to both HCs and the non-infarct region of the responsible artery (14). Patients with cerebral ischemia and stroke show abnormal iron accumulation in the brain, which may provide a novel perspective for comprehending the pathophysiological alterations of ischemic stroke.

The results of a study about computed tomographic perfusion and computed tomographic angiography suggest that advanced imaging variables (such as acute ischemic core volume, acute penumbra volume and collateral circulation grade) are strong predictors of prognosis in patients with AIS (39). Another study suggests collateral circulation grade is a prognostic indicator for patients who achieve recanalization, but not for patients who do not achieve recanalization (40). However, a review suggests that the low consistency of stroke trials with different imaging modalities may affect the development of new treatment strategies for AIS (41). A study found that oxygen extraction fraction map generated based on QSM can help identify the ischemic penumbra in patients with AIS, suggesting that advanced MRI has the potential to guide treatment choices for patients with AIS (42). A QSM-based study found that longitudinal changes in iron and myelination within ischemic lesions were associated with neurologic prognosis, and that increased iron concentrations within ischemic lesions were associated with less improvement in neurologic prognosis (43). Although the diagnostic accuracy of susceptibility values in CN and PUT has moderate predictive value in this study, enhanced use of advanced imaging biomarkers may help us find the underlying molecular-level pathophysiologic basis of AIS. Study of iron deposition in GM nuclei of patients with AIS may provide new ideas for prevention and therapeutic drug development of stroke. We will deeply explore the relationship between susceptibility values of deep GM nuclei and other imaging biomarkers about AIS as well as the prognosis of AIS in the future.

The CN and PUT form the dorsal striatum, which coordinates various functions including motor activity, motivation and learning (44). When focusing on functional connections, the PUT showed a high degree of co-activation with the primary motor cortex, whereas the CN showed a high degree of co-activation with the area of high-level cognitive functions (45). Previous studies have demonstrated a significant accumulation of iron in CN and PUT in patients with neurodegenerative diseases, such as Alzheimer’s disease, Parkinson’s disease and Wilson’s disease (46–49). Iron overload within the CN and PUT may contributes to iron-dependent cell death, and the neuronal death of the CN and PUT could result in neurological impairment and cognition deficit (23, 50). The result in the present study may provide a plausible explanation for motor and cognition deficit in patients with AIS. As a final note, our study has specifically focused on the susceptibility values of the head region of the CN, and do not focus on the body or tail.

AIS causes rapid neuronal damage and death in the central area of the infarction and in the surrounding hypoperfused region. In addition to this, cerebral ischemia also induces neuronal degeneration in distal regions which are connected to the area of ischemic core (51). For example, after cerebral infarction in the middle cerebral artery region, neuronal death, gliosis, and axonal degeneration were also found in the ipsilateral THA, SN, and distal pyramidal tract that outside the middle cerebral artery region (52). An animal study from rat models demonstrated significant atrophy and neurodegeneration in the ipsilateral SN after middle cerebral artery occlusion (53). Several days after the basal ganglia infarction, DWI showed degeneration in the ipsilateral SN of the patient (52). The SN plays an important role in motor control along with the basal ganglia, so degeneration of SN may be associated with poor functional or motor outcomes of the patient with AIS (54). Degeneration of SN cells and decreased dopamine synthesis are the main causes of Parkinson’s disease. A large population-based analysis found that cerebrovascular risk factors such as a history of stroke were strongly associated with a subsequent diagnosis of Parkinson’s disease (55). We hypothesized that stroke may cause degeneration of SN cells leading to Parkinson’s disease. Our findings revealed that the susceptibility values of SN showed lateral asymmetry in patients with AIS but not in HCs, suggesting that alterations of iron level may not necessarily be paralleled between left and right after the onset of stroke. Notably, conventional MRI including T1WI, T2WI, T2-FLAIR, and DWI in our experiments did not reveal any abnormalities in SN signals in patients with AIS. Therefore, we hypothesized that the change in iron content in SN may have occurred prior to neuronal degeneration and cytotoxic edema. It should be noted that whether lateral asymmetry of the SN is related to the site of cerebral infarction require further examination in the future.

This study had several limitations. First, the number of participants was relatively small. A larger sample size, especially patients with AIS, is required to validate the finding of the current study. Second, the current study focused on the brain iron changes in the GM nuclei, while the study of susceptibility values in white matter and cerebral cortex may also contribute to the understanding of AIS. Third, as a cross-sectional study, this research still cannot be used to accurately speculate on the cause-and-effect relationship between abnormal iron accumulation and AIS. Fourth, the lack of measurements of ROI volume is a limitation, as it may affect the evaluation of susceptibility value and the experimental results. Nevertheless, we ensured that ROI was evaluated in similar slices of different participant as much as possible to reduce the impact on the experimental results. And then, the average susceptibility value and standard deviation of each ROI was evaluated on three consecutive slices to improve the accuracy of the experiment. In future studies, we will include ROI volume as a covariate in statistical models to improve the reliability and validity of the study.

## 5 Conclusion

As measured by QSM, iron levels in the bilateral CN and PUT were significantly increased in patients with AIS. Abnormal iron accumulation in the basal ganglia region might be related to the pathophysiological changes of AIS. These findings may open the door to in-depth study of the pathophysiological mechanisms of AIS.

## Data Availability

The raw data supporting the conclusions of this article will be made available by the authors, without undue reservation.

## References

[1] Schadlich IS Winzer R Stabernack J Tolosa E Magnus T Rissiek B. The role of the ATP-adenosine axis in ischemic stroke. Semin Immunopathol. (2023) 45:347–65. doi: 10.1007/s00281-023-00987-3, PMID: 36917241 PMC10279578

[2] Donnan GA Fisher M Macleod M Davis SM. Stroke. Lancet. (2008) 371:1612–23. doi: 10.1016/S0140-6736(08)60694-7 18468545

[3] Mendelson SJ Prabhakaran S. Diagnosis and Management of Transient Ischemic Attack and Acute Ischemic Stroke. JAMA. (2021) 325:1088. doi: 10.1001/jama.2020.26867 33724327

[4] Oku N Kashiwagi T Hatazawa J. Nuclear neuroimaging in acute and subacute ischemic stroke. Ann Nucl Med. (2010) 24:629–38. doi: 10.1007/s12149-010-0421-7, PMID: 20953742

[5] Eskreis-Winkler S Zhang Y Zhang J Liu Z Dimov A Gupta A. The clinical utility of QSM: disease diagnosis, medical management, and surgical planning. NMR Biomed. (2017) 30:3668. doi: 10.1002/nbm.3668 27906525

[6] Otani S Fushimi Y Iwanaga K Tomotaki S Shimotsuma T Nakajima S. Evaluation of deep gray matter for early brain development using quantitative susceptibility mapping. Eur Radiol. (2023) 33:4488–99. doi: 10.1007/s00330-022-09267-4, PMID: 36418626

[7] Crichton RR Dexter DT Ward RJ. Brain iron metabolism and its perturbation in neurological diseases. J Neural Transm (Vienna). (2011) 118:301–14. doi: 10.1007/s00702-010-0470-z, PMID: 20809066

[8] Lotharius J Brundin P. Pathogenesis of Parkinson's disease: dopamine, vesicles and alpha-synuclein. Nat Rev Neurosci. (2002) 3:932–42. doi: 10.1038/nrn983 12461550

[9] Langkammer C Schweser F Krebs N Deistung A Goessler W Scheurer E. Quantitative susceptibility mapping (QSM) as a means to measure brain iron? A post mortem validation study. Neuroimage. (2012) 62:1593–9. doi: 10.1016/j.neuroimage.2012.05.049, PMID: 22634862 PMC3413885

[10] Probst J Rohner M Zahn M Piccirelli M Pangalu A Luft A. Quantitative susceptibility mapping in ischemic stroke patients after successful recanalization. Sci Rep. (2021) 11:16038. doi: 10.1038/s41598-021-95265-3, PMID: 34362957 PMC8346586

[11] Vinayagamani S Sheelakumari R Sabarish S Senthilvelan S Ros R Thomas B. Quantitative susceptibility mapping: technical considerations and clinical applications in neuroimaging. J Magn Reson Imaging. (2021) 53:23–37. doi: 10.1002/jmri.27058, PMID: 31951057

[12] Du L Zhao Z Cui A Zhu Y Zhang L Liu J. Increased Iron deposition on brain quantitative susceptibility mapping correlates with decreased cognitive function in Alzheimer's disease. ACS Chem Neurosci. (2018) 9:1849–57. doi: 10.1021/acschemneuro.8b00194, PMID: 29722955

[13] Reichenbach JR. The future of susceptibility contrast for assessment of anatomy and function. NeuroImage. (2012) 62:1311–5. doi: 10.1016/j.neuroimage.2012.01.004 22245644

[14] Yang J Lv M Han L Li Y Liu Y Guo H. Evaluation of brain iron deposition in different cerebral arteries of acute ischaemic stroke patients using quantitative susceptibility mapping. Clin Radiol. (2024) 79:e592–8. doi: 10.1016/j.crad.2024.01.007, PMID: 38320942

[15] Kim HG Park S Rhee HY Lee KM Ryu CW Rhee SJ. Quantitative susceptibility mapping to evaluate the early stage of Alzheimer's disease. Neuroimage Clin. (2017) 16:429–38. doi: 10.1016/j.nicl.2017.08.019, PMID: 28879084 PMC5577408

[16] Uchida Y Kan H Sakurai K Arai N Kato D Kawashima S. Voxel-based quantitative susceptibility mapping in Parkinson's disease with mild cognitive impairment. Mov Disord. (2019) 34:1164–73. doi: 10.1002/mds.27717, PMID: 31091347

[17] Uchida Y Kan H Sakurai K Horimoto Y Hayashi E Iida A. APOE varepsilon 4 dose associates with increased brain iron and beta-amyloid via blood-brain barrier dysfunction. J Neurol Neurosurg Psychiatry. (2022) 93:772–8. doi: 10.1136/jnnp-2021-328519 35483916

[18] Uchida Y Kan H Sakurai K Inui S Kobayashi S Akagawa Y. Magnetic susceptibility associates with dopaminergic deficits and cognition in Parkinson's disease. Mov Disord. (2020) 35:1396–405. doi: 10.1002/mds.28077, PMID: 32369660

[19] Uchida Y Kan H Sakurai K Oishi K Matsukawa N. Quantitative susceptibility mapping as an imaging biomarker for Alzheimer's disease: the expectations and limitations. Front Neurosci. (2022) 16:938092. doi: 10.3389/fnins.2022.938092, PMID: 35992906 PMC9389285

[20] Xu M Guo Y Cheng J Xue K Yang M Song X. Brain iron assessment in patients with first-episode schizophrenia using quantitative susceptibility mapping. Neuroimage Clin. (2021) 31:102736. doi: 10.1016/j.nicl.2021.102736, PMID: 34186296 PMC8254125

[21] Yan Z Liu H Chen X Zheng Q Zeng C Zheng Y. Quantitative susceptibility mapping-derived Radiomic features in discriminating multiple sclerosis from Neuromyelitis Optica Spectrum disorder. Front Neurosci. (2021) 15:765634. doi: 10.3389/fnins.2021.765634, PMID: 34924934 PMC8678528

[22] Campos-Escamilla C. The role of transferrins and iron-related proteins in brain iron transport: applications to neurological diseases. Adv Protein Chem Struct Biol. (2021) 123:133–62. doi: 10.1016/bs.apcsb.2020.09.002 33485481

[23] Du L Zhao Z Liu X Chen Y Gao W Wang Y. Alterations of Iron level in the bilateral basal ganglia region in patients with middle cerebral artery occlusion. Front Neurosci. (2020) 14:608058. doi: 10.3389/fnins.2020.608058, PMID: 33551726 PMC7859276

[24] Dusek P Hofer T Alexander J Roos PM Aaseth JO. Cerebral Iron deposition in neurodegeneration. Biomol Ther. (2022) 12:714. doi: 10.3390/biom12050714, PMID: 35625641 PMC9138489

[25] Tuo QZ Lei P Jackman KA Li XL Xiong H Li XL. Tau-mediated iron export prevents ferroptotic damage after ischemic stroke. Mol Psychiatry. (2017) 22:1520–30. doi: 10.1038/mp.2017.171 28886009

[26] Xia S Utriainen D Tang J Kou Z Zheng G Wang X. Decreased oxygen saturation in asymmetrically prominent cortical veins in patients with cerebral ischemic stroke. Magn Reson Imaging. (2014) 32:1272–6. doi: 10.1016/j.mri.2014.08.012, PMID: 25131626

[27] Li X Jin D Zhu Y Liu L Qiao Y Qian Y. Quantitative susceptibility mapping to evaluate brain iron deposition and its correlation with physiological parameters in hypertensive patients. Ann Transl Med. (2021) 9:1582. doi: 10.21037/atm-21-5170, PMID: 34790788 PMC8576670

[28] Wang W Jiang B Sun H Ru X Sun D Wang L. Prevalence, incidence, and mortality of stroke in China: results from a Nationwide population-based survey of 480 687 adults. Circulation. (2017) 135:759–71. doi: 10.1161/CIRCULATIONAHA.116.025250 28052979

[29] Mao H Dou W Chen K Wang X Wang X Guo Y. Evaluating iron deposition in gray matter nuclei of patients with unilateral middle cerebral artery stenosis using quantitative susceptibility mapping. Neuroimage Clin. (2022) 34:103021. doi: 10.1016/j.nicl.2022.103021, PMID: 35500369 PMC9065429

[30] Salvador GA. Iron in neuronal function and dysfunction. Biofactors. (2010) 36:103–10. doi: 10.1002/biof.80 20232345

[31] Selim MH Ratan RR. The role of iron neurotoxicity in ischemic stroke. Ageing Res Rev. (2004) 3:345–53. doi: 10.1016/j.arr.2004.04.001, PMID: 15231241

[32] Stankiewicz J Panter SS Neema M Arora A Batt CE Bakshi R. Iron in chronic brain disorders: imaging and neurotherapeutic implications. Neurotherapeutics. (2007) 4:371–86. doi: 10.1016/j.nurt.2007.05.006, PMID: 17599703 PMC1963417

[33] Hirschhorn T Stockwell BR. The development of the concept of ferroptosis. Free Radic Biol Med. (2019) 133:130–43. doi: 10.1016/j.freeradbiomed.2018.09.043, PMID: 30268886 PMC6368883

[34] Harrison AV Lorenzo FR McClain DA. Iron and the pathophysiology of diabetes. Annu Rev Physiol. (2023) 85:339–62. doi: 10.1146/annurev-physiol-022522-102832, PMID: 36137277 PMC10161568

[35] Hilton C Sabaratnam R Drakesmith H Karpe F. Iron, glucose and fat metabolism and obesity: an intertwined relationship. Int J Obes. (2023) 47:554–63. doi: 10.1038/s41366-023-01299-0, PMID: 37029208 PMC10299911

[36] Savarese G von Haehling S Butler J Cleland JGF Ponikowski P Anker SD. Iron deficiency and cardiovascular disease. Eur Heart J. (2023) 44:14–27. doi: 10.1093/eurheartj/ehac569, PMID: 36282723 PMC9805408

[37] Xi X Wu Q Wang X Sun X Yu G Jiang L. The association between iron metabolism with the change of blood pressure and risk of hypertension: a large cross-sectional study. J Trace Elem Med Biol. (2023) 79:127193. doi: 10.1016/j.jtemb.2023.127193, PMID: 37269648

[38] Mao H Dou W Wang X Chen K Wang X Guo Y. Iron deposition in gray matter nuclei of patients with intracranial artery stenosis: a quantitative susceptibility mapping study. Front Neurol. (2021) 12:785822. doi: 10.3389/fneur.2021.785822, PMID: 35069414 PMC8766754

[39] Bivard A Levi C Lin L Cheng X Aviv R Spratt NJ. Validating a predictive model of acute advanced imaging biomarkers in ischemic stroke. Stroke. (2017) 48:645–50. doi: 10.1161/STROKEAHA.116.015143 28104836

[40] Tong E Patrie J Tong S Evans A Michel P Eskandari A. Time-resolved CT assessment of collaterals as imaging biomarkers to predict clinical outcomes in acute ischemic stroke. Neuroradiology. (2017) 59:1101–9. doi: 10.1007/s00234-017-1914-z, PMID: 28864854

[41] Harston GW Rane N Shaya G Thandeswaran S Cellerini M Sheerin F. Imaging biomarkers in acute ischemic stroke trials: a systematic review. AJNR Am J Neuroradiol. (2015) 36:839–43. doi: 10.3174/ajnr.A4208 25634718 PMC5524169

[42] Uchida Y Kan H Inoue H Oomura M Shibata H Kano Y. Penumbra detection with oxygen extraction fraction using magnetic susceptibility in patients with acute ischemic stroke. Front Neurol. (2022) 13:752450. doi: 10.3389/fneur.2022.752450, PMID: 35222239 PMC8873150

[43] Uchida Y Kan H Kano Y Onda K Sakurai K Takada K. Longitudinal changes in Iron and myelination within ischemic lesions associate with neurological outcomes: a pilot study. Stroke. (2024) 55:1041–50. doi: 10.1161/STROKEAHA.123.044606, PMID: 38269537

[44] De Deurwaerdere P Gaetani S Vaughan RA. Old neurochemical markers, new functional directions?: an editorial for 'Distinct gradients of various neurotransmitter markers in caudate nucleus and putamen of the human brain' on page 650. J Neurochem. (2020) 152:623–6. doi: 10.1111/jnc.14929, PMID: 31917872

[45] Grahn JA Parkinson JA Owen AM. The cognitive functions of the caudate nucleus. Prog Neurobiol. (2008) 86:141–55. doi: 10.1016/j.pneurobio.2008.09.004 18824075

[46] Nathoo N Gee M Nelles K Burt J Sun H Seres P. Quantitative susceptibility mapping changes relate to gait issues in Parkinson's disease. Can J Neurol Sci. (2023) 50:853–60. doi: 10.1017/cjn.2022.316 36351571

[47] Rao IY Hanson LR Johnson JC Rosenbloom MH Frey WH 2nd. Brain glucose Hypometabolism and Iron accumulation in different brain regions in Alzheimer's and Parkinson's diseases. Pharmaceuticals. (2022) 15:551. doi: 10.3390/ph15050551, PMID: 35631378 PMC9143620

[48] Yang A Du L Gao W Liu B Chen Y Wang Y. Associations of cortical iron accumulation with cognition and cerebral atrophy in Alzheimer's disease. Quant Imaging Med Surg. (2022) 12:4570–86. doi: 10.21037/qims-22-7, PMID: 36060596 PMC9403583

[49] Yuan XZ Li GY Chen JL Li JQ Wang XP. Paramagnetic metal accumulation in the deep gray matter nuclei is associated with neurodegeneration in Wilson's disease. Front Neurosci. (2020) 14:573633. doi: 10.3389/fnins.2020.573633 33041766 PMC7525019

[50] Wan J Ren H Wang J. Iron toxicity, lipid peroxidation and ferroptosis after intracerebral haemorrhage. Stroke Vasc Neurol. (2019) 4:93–5. doi: 10.1136/svn-2018-000205, PMID: 31338218 PMC6613877

[51] Rodriguez-Grande B Blackabey V Gittens B Pinteaux E Denes A. Loss of substance P and inflammation precede delayed neurodegeneration in the substantia nigra after cerebral ischemia. Brain Behav Immun. (2013) 29:51–61. doi: 10.1016/j.bbi.2012.11.017, PMID: 23232501

[52] Zhang J Zhang Y Xing S Liang Z Zeng J. Secondary neurodegeneration in remote regions after focal cerebral infarction: a new target for stroke management? Stroke. (2012) 43:1700–5. doi: 10.1161/STROKEAHA.111.632448, PMID: 22492515

[53] Nakanishi H Tamura A Kawai K Kawai K Yamamoto K Yamamoto K. Electrophysiological studies of rat substantia nigra neurons in an in vitro slice preparation after middle cerebral artery occlusion. Neuroscience. (1997) 77:1021–8. doi: 10.1016/s0306-4522(96)00555-6 9130783

[54] Lee H Lee K Kim YD Nam HS Lee HS Cho S. Association between substantia nigra degeneration and functional outcome in patients with basal ganglia infarction. Eur J Neurol. (2024) 31:e16111. doi: 10.1111/ene.16111, PMID: 37903090 PMC10841447

[55] Kummer BR Diaz I Wu X Aaroe AE Chen ML Iadecola C. Associations between cerebrovascular risk factors and parkinson disease. Ann Neurol. (2019) 86:572–81. doi: 10.1002/ana.25564 31464350 PMC6951811

